# Endoscopic Removal of a Displaced Dental Implant From the Tail of the Inferior Nasal Concha

**DOI:** 10.7759/cureus.104341

**Published:** 2026-02-26

**Authors:** Panagiotis Giasimakopoulos, Dimitris Tatsis, Chrysoula Vardaxi, Stylianos Varagkas, Konstantinos Paraskevopoulos

**Affiliations:** 1 Oral and Maxillofacial Surgery, General Hospital "George Papanikolaou" Aristotle University of Thessaloniki, Thessaloniki, GRC; 2 Otorhinolaryngology-Head and Neck Surgery, General Hospital "George Papanikolaou" Aristotle University of Thessaloniki, Thessaloniki, GRC

**Keywords:** case report, dental implant displacement, endoscopic removal, inferior nasal concha, nasal cavity

## Abstract

Accidental displacement of dental implants into adjacent anatomical cavities is a rare but clinically significant complication of implant surgery. Migration into the nasal cavity, particularly into posterior regions such as the tail of the inferior nasal concha, is exceedingly uncommon and sparsely documented in the literature. Prompt radiographic evaluation and coordinated interdisciplinary management are essential in preventing secondary complications and determining the most appropriate treatment approach. We present the case of a 65-year-old woman who was referred after an accidental displacement of a dental implant placed at site #23. Computed tomography revealed an 11 × 4 mm dental implant lodged beneath the left inferior nasal concha, associated with hemorrhagic collection in the ipsilateral nasal cavity and maxillary sinus. A prior surgical retrieval attempt was unsuccessful. The implant was subsequently removed endoscopically under local anesthesia after consulting with the otorhinolaryngology team. The implant was visualized and extracted intact without additional mucosal injury. The postoperative course was uneventful, and at one-week follow-up, the patient reported complete resolution of nasal obstruction, with endoscopy confirming normal mucosal recovery. This case highlights a rare anatomical location of implant migration and demonstrates the effectiveness of minimally invasive endoscopic removal. Early imaging, careful assessment of sinonasal anatomy, and interdisciplinary management between oral and maxillofacial surgery and ear, nose, and throat specialists are fundamental for ensuring optimal outcomes.

## Introduction

Dental implant therapy is a reliable and widely practiced modality in oral rehabilitation; however, complications may arise when implants migrate into adjacent anatomical spaces. The maxillary sinus is the most frequently affected region due to its close proximity to the posterior maxilla, reduced residual bone height, and sinus pneumatization, which may compromise primary stability [1,2]. In contrast, migration into the nasal cavity is rare and has been described mainly in isolated reports involving perforation of the nasal floor or protrusion into the anterior nasal cavity [3-8]. Clinical manifestations range from asymptomatic presentation to nasal obstruction, rhinorrhea, or recurrent sinusitis, depending on implant location [5,6].

Proposed mechanisms include intraoperative perforation during osteotomy preparation, insufficient bone support, peri-implant bone loss, and anatomical variations facilitating posterior displacement [4-9]. Imaging studies have highlighted inferior meatus pneumatization and posterior nasal cavity extension as potential predisposing factors [7,8,10].

Endoscopic nasal surgery is currently the preferred minimally invasive retrieval method due to improved visualization and reduced morbidity compared with traditional open approaches such as the Caldwell-Luc procedure [9-13]. To our knowledge, migration of a dental implant into the tail of the inferior nasal concha-the posterior-most segment of the turbinate-has not been previously reported [3-8]. We present a case of posterior implant migration from site #23 into the tail of the inferior nasal concha following an unsuccessful retrieval attempt, emphasizing the importance of early imaging and interdisciplinary collaboration for optimal management.

## Case presentation

A 65-year-old woman was referred to the Department of Oral and Maxillofacial Surgery (OMFS) at the General Hospital “G. Papanikolaou,” Thessaloniki (tertiary center) for evaluation and management of an accidentally displaced dental implant placed two days earlier at site #23. The patient’s past medical history included type II diabetes mellitus (controlled under regular follow-up by her primary physician, with no history of diabetes-related complications), dyslipidemia, and hypothyroidism, which were well managed. She had undergone septorhinoplasty several years prior, with no residual functional complaints or chronic sinonasal pathology. She reported no drug allergies and had no history of recurrent sinusitis, nasal polyps, or chronic nasal obstruction prior to the current episode.

The patient recounted that during routine implant placement at the left maxillary central canine region (#23), the implant suddenly migrated superiorly and disappeared from the surgical field. According to the patient and the referring surgeon, the osteotomy had been completed without intraoperative complications until the moment of implant insertion, when the fixture unexpectedly lost primary stability and was no longer visualized. Immediate radiographs taken at the private practice suggested displacement. The patient was then referred to our OMFS department for further assessment and definitive management.

On arrival, the patient’s chief concern was the missing dental implant. She also reported the onset of mild left-sided nasal obstruction since the initial implantation attempt but denied epistaxis, nasal discharge, facial pain, or headache. She had no fever, chills, or systemic symptoms. She denied trismus, dysphagia, or intraoral discomfort and had been able to resume regular oral intake. She did not report any breathing difficulty and noted no change in her sense of smell. Importantly, she denied visual disturbances, diplopia, or periorbital swelling, and there were no symptoms suggestive of orbital involvement.

Clinical examination revealed a healthy-appearing woman in no acute distress. Extraoral inspection showed no facial asymmetry, swelling, or erythema. Palpation of the maxilla and midface demonstrated no tenderness or crepitus. The incision line appeared clean, without purulence, drainage, or evidence of dehiscence. The surrounding mucosa was of normal color and turgor. The mucobuccal fold was preserved, and there were no signs of oroantral communication such as air escape, bubbling, or fluid regurgitation. Mandibular opening was normal, with no trismus or deviation upon opening.

Anterior rhinoscopy demonstrated mild obstruction of the left nasal cavity, predominantly along the inferior meatus. The nasal mucosa was slightly edematous but without active bleeding, crusting, polyps, or purulent secretions. The septum appeared midline, with no perforation or acute deviation attributable to the displaced implant.

**Figure 1:**
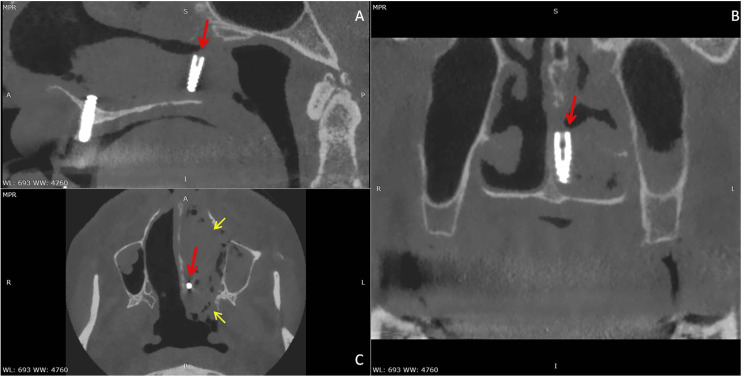
CT scan (A: sagittal, B: coronal, and C: axial views). A cylindrical, hyperdense foreign body measuring approximately 11 × 4 mm situated beneath the tail of the left inferior nasal concha, extending posteriorly toward the choana (red arrow). Evidence of hemorrhagic collection within the left nasal cavity and the ipsilateral maxillary sinus (yellow arrow). CT: computed tomography

**Figure 2:**
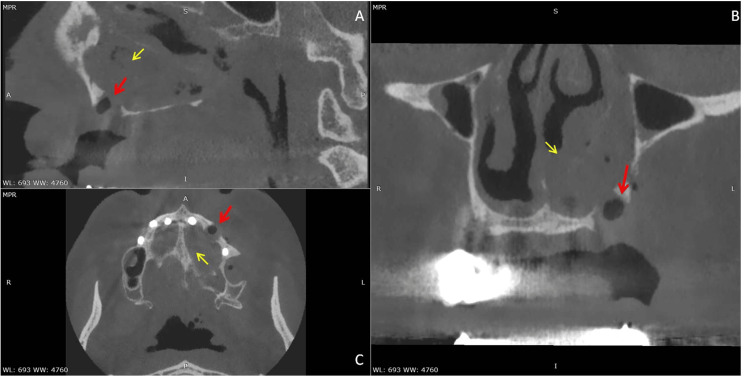
CT scan (A: sagittal, B: coronal, and C: axial views). Initial implant site, positioned approximately in the site of the upper left canine #23, communicating with the nasal cavity (red arrow). Evidence of hemorrhagic collection within the left nasal cavity and the ipsilateral maxillary sinus (yellow arrow). CT: computed tomography

Given the uncertainty regarding the implant’s exact location and the unsuccessful previous retrieval attempt, computed tomography (CT) of the paranasal sinuses was obtained. CT imaging revealed a cylindrical, hyperdense foreign body measuring approximately 11 × 4 mm situated beneath the tail of the left inferior nasal concha, extending posteriorly toward the choana (Figure 1). The implant lay in close proximity to the hard palate and nasal septum, without penetration into the nasopharynx. Surrounding the implant, there was evidence of hemorrhagic collection within the left nasal cavity and the ipsilateral maxillary sinus. Diffuse mucosal thickening was observed throughout the maxillary sinus and along the inferior meatus, consistent with reactive changes following the displacement (Figure 2). The boundaries of the inferior turbinate and nasal septum remained intact, with no bony fractures or displacement.

A multidisciplinary consultation with the otorhinolaryngology (ENT) team was promptly arranged. Given the implant’s posterior location in the nasal cavity and the limitations of the prior surgical approach, endoscopic transnasal removal under local anesthesia was recommended as the safest and least invasive option. Risks, benefits, and alternatives were discussed with the patient, who consented to the proposed management plan.

**Figure 3:**
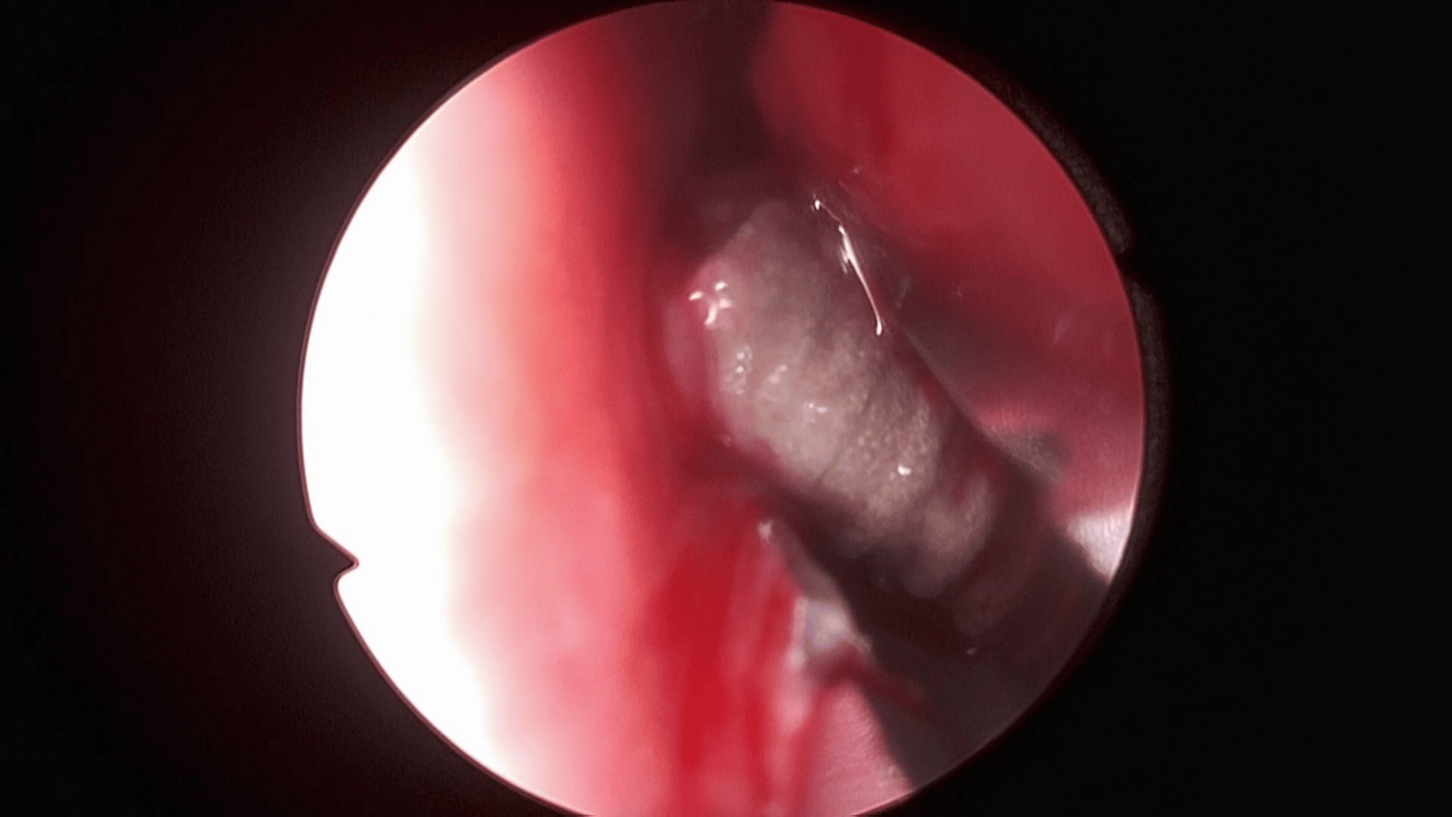
Endoscopic intraoperative view showing the displaced dental implant lodged beneath the left inferior nasal concha prior to removal.

An endoscopic removal of the displaced implant was performed under local anesthesia with lidocaine. A rigid nasal endoscope provided direct visualization of the left nasal cavity and the posterior inferior meatus. The implant was identified lodged beneath the tail of the inferior nasal concha, toward the nasopharyngeal region. Using delicate nasal forceps under endoscopic guidance, the implant was carefully mobilized and extracted intact, without causing additional mucosal injury or significant bleeding (Figure 3).

The retrieved implant was examined and found to be structurally intact and free of visible deformation. There was no evidence of surrounding granulation tissue or active infection at the retrieval site.

Postoperative management included analgesics, an antimicrobial regimen (amoxicillin/clavulanate 1.2 g three times daily for five days), and topical nasal decongestants to reduce mucosal edema and minimize the risk of secondary sinonasal infection. Topical nasal decongestants were prescribed for 10 days to reduce mucosal edema and support sinonasal drainage.

The patient’s postoperative course was uneventful. At the one-week follow-up visit, she reported immediate and sustained improvement of nasal breathing, with complete relief of left-sided nasal obstruction and absence of nasal discharge or facial pain. Follow-up nasal endoscopy showed normal healing of the mucosa at the site of implant lodgment, without residual hemorrhage, infection, or granulation tissue. No septal perforation, turbinate damage, or adhesions were observed.

Over the subsequent follow-up period, no adverse events, recurrent symptoms, or delayed complications were reported. At the two-month telephone follow-up, the patient remained asymptomatic, reporting no nasal obstruction, discharge, facial pain, or functional limitation. The patient expressed high satisfaction with the minimally invasive management and the rapid resolution of her symptoms.

## Discussion

Accidental migration of dental implants into the sinonasal region represents an infrequent but clinically significant complication of implant surgery. The maxillary sinus is the most commonly involved site due to its anatomical relationship with the posterior maxilla, reduced residual bone height, and frequent sinus pneumatization following tooth loss [1,2]. In contrast, intranasal displacement is rare and has been described mainly in isolated reports involving perforation of the nasal floor or protrusion into the anterior nasal cavity [3-8]. Migration into the posterior nasal cavity, particularly beneath the tail of the inferior nasal concha as observed in the present case, has not been previously documented, underscoring its exceptional rarity [3-8].

Several etiological factors may contribute to implant migration into sinonasal structures. Insufficient bone density or height can compromise primary stability [1,2], rendering the implant susceptible to displacement during insertion or under functional loading [1,2,14]. Over-preparation of the osteotomy site or excessive insertion torque may weaken the bone-implant interface and facilitate superior migration [4,5]. Peri-implant inflammatory bone loss has also been implicated in reducing surrounding bone support and allowing gradual displacement toward adjacent cavities [4,9]. In addition, anatomical variations of the nasal cavity and maxillary sinus play a critical role. Inferior meatus pneumatization, posterior extension of the nasal cavity, and turbinate morphology variations may create a pathway of least resistance for implant displacement [7,8,11].

In the present case, implant displacement likely resulted from insufficient primary stability in the anterior maxilla, potentially related to limited local bone support and close proximity to the nasal cavity. Although the patient had no known chronic sinonasal pathology, imaging demonstrated communication between the implant site and the nasal cavity, suggesting reduced vertical bone support. This case highlights the importance of thorough preoperative assessment of residual bone height and nasal floor anatomy, particularly in anatomically borderline regions where subtle variations may increase the risk of superior migration.

Clinical presentation varies according to implant location. Some patients remain asymptomatic, with displacement detected incidentally on imaging [5,6], whereas others present with nasal obstruction, rhinorrhea, halitosis, facial pain, or recurrent sinusitis secondary to mucosal irritation or impaired drainage [5-7]. Spontaneous expulsion through the nasal cavity has also been reported [6]. In our patient, only mild unilateral nasal obstruction was noted despite CT evidence of hemorrhagic collection and mucosal thickening. This underscores the importance of investigating any unexplained intraoperative loss of implant stability, even in the absence of significant sinonasal symptoms.

Accurate imaging is essential for precise localization and surgical planning. CT remains the gold standard, providing detailed information regarding implant position, orientation, and its relationship to adjacent structures [9,10]. In this case, CT enabled identification of the implant at the tail of the inferior turbinate, a location inaccessible via the initial surgical approach, and demonstrated associated reactive changes. Cone-beam CT (CBCT) has also been highlighted as a valuable tool for identifying anatomical variations that may predispose to implant protrusion [7,8,11].

Historically, open approaches such as the Caldwell-Luc procedure were used for the retrieval of maxillary sinus foreign bodies but are associated with increased morbidity, including infraorbital nerve injury, postoperative pain, and disruption of normal sinus physiology [1,2,15]. In contemporary practice, endoscopic nasal surgery is the preferred treatment modality for displaced implants due to superior visualization, targeted access, reduced morbidity, and faster recovery [9-13]. In the present case, the endoscopic approach permitted direct visualization and atraumatic removal under local anesthesia.

The favorable postoperative outcome aligns with prior reports demonstrating the safety and effectiveness of endoscopic retrieval of sinonasal foreign bodies [9-13]. In our patient, rapid symptom resolution and normal endoscopic findings were observed, with no evidence of turbinate injury or persistent inflammation. At the two-month telephone follow-up, the patient remained asymptomatic without recurrence of nasal obstruction, discharge, or facial pain.

Preventive strategies remain essential. Thorough preoperative evaluation of bone quality, residual bone height, and sinonasal anatomy using CT or CBCT is critical in identifying potential risk factors [1,2,7,8,11,14]. Awareness of anatomical variations-particularly inferior meatus pneumatization and posterior nasal cavity extension-should guide implant trajectory planning. Controlled drilling techniques, avoidance of over-preparation, and careful assessment of primary stability are fundamental in preventing displacement. In compromised cases, adjunctive procedures such as sinus floor elevation or guided bone regeneration may be considered [14,15].

Early recognition and prompt multidisciplinary management are crucial to prevent complications such as chronic sinusitis, persistent obstruction, or further migration [3,16]. In this case, coordinated collaboration between OMFS and otorhinolaryngology teams enabled accurate diagnosis and successful minimally invasive treatment with an excellent clinical outcome [17].

## Conclusions

The present case highlights a rare posterior migration of a dental implant from the anterior maxilla into the tail of the inferior nasal concha, a location not previously described in the literature. Early recognition of implant displacement, even in the presence of minimal symptoms, is critical, and CT remains essential for accurate localization and treatment planning. Endoscopic transnasal removal provided direct visualization, precise access to the posterior nasal cavity, and minimal postoperative morbidity, resulting in complete symptom resolution.

This case emphasizes the importance of careful anatomical assessment in regions where bone support may be limited and subtle sinonasal variations may exist. Although implant displacement into the posterior nasal cavity is exceedingly uncommon, individualized evaluation of bone conditions and anatomical proximity to the nasal floor is advisable. Successful management in this case was achieved through timely interdisciplinary collaboration between OMFS and otorhinolaryngology teams, enabling accurate diagnosis and minimally invasive treatment.

## References

[1] Annibali S, Ripari M, LA Monaca G, Tonoli F, Cristalli MP (2008). Local complications in dental implant surgery: prevention and treatment. Oral Implantol (Rome).

[2] Jeong KI, Kim SG, Oh JS, Jeong MA (2014). Displaced implants into maxillary sinus: report of cases. Implant Dent.

[3] Li S, Xing Z, Yu L (2020). Accidental migration of a dental implant into the nasal cavity. J Int Med Res.

[4] Safi Y, Mortazavi H, Sadeghian A, Hazrati P (2022). Accidental displacement of a dental implant into the nasal cavity: report of a rare case. Clin Case Rep.

[5] Sanchis JM, Díaz JM (2021). Accidental migration of dental implant into the nasal cavity: spontaneous expulsion through the nose. J Clin Exp Dent.

[6] van de Loo S, Kessler P, Lethaus B (2013). Spontaneous transmaxillary-transnasal implant loss: a case report. Implant Dent.

[7] Yeom HG, Huh KH, Yi WJ, Heo MS, Lee SS, Choi SC, Kim JE (2023). Nasal cavity perforation by implant fixtures: case series with emphasis on panoramic imaging of nasal cavity extending posteriorly. Head Face Med.

[8] Park WB, Kim YJ, Kang KL, Lim HC, Han JY (2020). Long-term outcomes of the implants accidentally protruding into nasal cavity extended to posterior maxilla due to inferior meatus pneumatization. Clin Implant Dent Relat Res.

[9] Yoon SH, Jung S, Kang T, Yang HC (2019). Accidental swallowing of dental implant: complication of transnasal endoscopic removal from maxillary sinus. J Oral Implantol.

[10] Park WB, Kim YJ, Han JY, Park JS, Kang P (2021). Radiographic and nasal endoscopic observation of accidentally perforated anterior nasal cavity with dental implants: case reports with 5-23 years of follow-up. J Oral Implantol.

[11] Safadi A, Ungar OJ, Oz I, Koren I, Abergel A, Kleinman S (2020). Endoscopic sinus surgery for dental implant displacement into the maxillary sinus-a retrospective clinical study. Int J Oral Maxillofac Surg.

[12] Lubbe DE, Aniruth S, Peck T, Liebenberg S (2008). Endoscopic transnasal removal of migrated dental implants. Br Dent J.

[13] Nakamura N, Mitsuyasu T, Ohishi M (2004). Endoscopic removal of a dental implant displaced into the maxillary sinus: technical note. Int J Oral Maxillofac Surg.

[14] Felisati G, Chiapasco M, Lozza P (2013). Sinonasal complications resulting from dental treatment: outcome-oriented proposal of classification and surgical protocol. Am J Rhinol Allergy.

[15] Kadoya K, Kunisada Y, Obata K, Takakura H, Ogawa T, Ibaragi S (2025). A case of a metal foreign object remaining in the maxillary bone for an extended period: a case report. Clin Case Rep.

[16] Allevi F, Fadda GL, Rosso C (2022). Treatment of sinusitis following dental implantation: a systematic review and meta-analysis. Am J Rhinol Allergy.

[17] Chiapasco M, Felisati G, Maccari A, Borloni R, Gatti F, Di Leo F (2009). The management of complications following displacement of oral implants in the paranasal sinuses: a multicenter clinical report and proposed treatment protocols. Int J Oral Maxillofac Surg.

